# Current clinical management of constitutional delay of growth and puberty

**DOI:** 10.1186/s13052-022-01242-5

**Published:** 2022-03-24

**Authors:** Rossella Gaudino, Gianpaolo De Filippo, Elena Bozzola, Manuela Gasparri, Mauro Bozzola, Alberto Villani, Giorgio Radetti

**Affiliations:** 1 Department of Surgical Sciences, Dentistry, Gynecology and Pediatrics, Pediatric Division, University of Verona, Verona, Italy; 2 Assistance Publique-Hôpitaux de Paris, Hôpital Robert Debré, Service d’Endocrinologie et Diabétologie Pédiatrique, Paris, France; 3 French Clinical Research Group in Adolescent Medicine and Health, Paris, France; 4 Pediatric Unit, IRCCS Bambino Gesù Children Hospital, Rome, Italy; 5 Department of Pediatrics, San Paolo Hospital, Milan, Italy; 6 University of Pavia, Pavia, Italy; 7 Provincia Autonoma di Bolzano, Bolzano, Italy

**Keywords:** Puberty, Delayed puberty, Bone age, Growth, Constitutional delay of growth and puberty, Hypogonadotropic hypogonadism

## Abstract

**Background:**

Constitutional delay of growth and puberty (CDGP) is classified as the most frequent cause of delayed puberty (DP). Finding out the etiology of DP during first evaluation may be a challenge. In details, pediatricians often cannot differentiate CDGP from permanent hypogonadotropic hypogonadism (PHH), with definitive diagnosis of PHH awaiting lack of puberty by age 18 yr. Neverthless, the ability in providing a precise and tempestive diagnosis has important clinical consequences.

**Main text:**

A growth failure in adolescents with CDGP may occur until the onset of puberty; after that the growth rate increases with rapidity. Bone age is typically delayed. CDGP is generally a diagnosis of exclusion. Nevertheless, other causes of DP must be evaluated. A family history including timing of puberty in the mother and in the father as well as physical examination may givee information on the cause of DP. Patients with transient delay in hypothalamic-pituitary-gonadal axis maturation due to associated conditions, such as celiac disease, inflammatory bowel diseases, kidney insufficiency and anorexia nervosa, may experience a functional hypogonadotropic hypogonadism. PHH revealing testosterone or estradiol low serum values and reduced FSH and LH levels may be connected to abnormalities in the central nervous system. So, magnetic resonance imaging is required in order to exclude either morphological alterations or neoplasia. If the adolescent with CDGP meets psychological difficulties, treatment is recommended.

**Conclusion:**

Even if CDGP is considered a variant of normal growth rather than a disease, short stature and retarded sexual development may led to psychological problems, sometimes associated to a poor academic performance. A prompt and precise diagnosis has an important clinical outcome. Aim of this mini-review is throwing light on management of patients with CDGP, emphasizing the adolescent diagnosis and trying to answer all questions from paediatricians.

## Background

The most common cause of both short stature and pubertal delay ^is^ constitutional delay of growth and puberty (CDGP), which affects over 2% of adolescents, mainly boys. These subjects experience a slowdown in the linear growth within the first 3 years of life, followed by a regular growth even if lower than their peers in the subsequent years. At the average age of puberty, the height begins to move further from the growth curve because of delay in the pubertal growth spurt onset. The CDGP subjects present a spontaneous catch-up growth, an onset of puberty, and a pubertal growth spurt later than average but often fail to achieve the genetic target height [1, 2]. Although CDGP is a variant of normal growth rather than a disease, short stature and retarded sexual development may contribute to psychological difficulties, sometimes associated to with poor academic performance. Patients with CDGP have a family history of delayed puberty in 50-75% of cases. The aetiology is not clear. The key genetic regulators in self-limited delayed of puberty (DP) are largely unknown. Via next generation sequencing, mutations in the following genes including *HS6ST1*, *GNRHR*, *IL17RD*, *SEMA3A*, *TACR3* and *TAC3,* have been found in patients with DP and spontaneous onset of puberty [3]. You may speculate that a single deleterious mutation lead to a phenotype of DP, whilst two or more mutations may be required to cause absent puberty, for example in congenital hypogonadotropic hypogonadism (HH) [4].

## Main text

The clinician who consults a subject with both short stature and inadequate virilisation may suspect a condition of a delay of growth and puberty. He must keep in mind some main points. First of all, DP is considered the very end of the distribution of the normal timing of puberty, not a pathology. In Caucasians, DP may be defined as the absence of breast bud at 13 years of age in the female or a time gap of more than 5 years from the breast buds to menarche, or no menstruation by age 16 years [5]. As for the male gender, DP may be defined as lack of increase in testicular volume > 4 ml at 14 years of age or a time gap of more than 5 years from the start to the end of genital growth [6].

### Diagnostic approach

To make a correct diagnosis the clinician should follow a simple *iter* [7]. After a detailed medical history and a correct auxological evaluation including height, weight, growth rate and evaluation of pubertal development and its progression, a clinical examination aimed at identifying particular signs should be done. CDGP occurs in healthy adolescents with an height reduced considering chronological age but appropriate considering bone age and pubertal development, which are delayed as well. In particular, CDGP adolescents may show a peripubertal deceleration of growth velocity (slowing down) associated with both delayed pubertal development and bone maturation [8]. The diagnosis requires the exclusion other causes of pubertal delay mainly organic, genetic and nutritional diseases, such as intestinal malabsorption, subclinical hypothyroidism or cystic fibrosis. A close relation between the stages of pubertal development and the start of pubertal spurt has been suggested. The pubertal growth spurt is usually observed in girls at the first stages of breast, approximately at Tanner II and III stage, while in boys when testicular volume reaches 10-12 ml. Loss of the normal harmony of growth and puberty could suggest an endocrinopathy. However, the normal consonance of growth and pubertal development is characteristic of the subjects with CDGP who do not require specific investigation. Distinguishing between CDGP and HH is especially difficult during initial evaluation because adolescents with these aetiologies are often prepubertal. Neither baseline nor GnRH stimulated gonadotropin levels are capable to differentiate CDGP from permanent hypogonadotropic hypogonadism [7]. Recently, new developments raise the availability of differentiate CDGP from CHH in the clinical setting [9]. The FSH-stimulated inhibin B has been shown to correctly differentiate pubertal delay from a hypogonadoptrophic hypogonadism; however further studies are need to confirm this interesting point [10]. A family history of DP strongly suggests a condition of CDGP (observed in 50–75%). The main characteristics of the three groups are summarized in Table 1. (Table 1) Generally, delayed puberty is a transient condition and has a good prognosis both in terms of final height and of reproductive ability. As growth potential is related to the degree of epiphyseal maturation, bone age delay allows a final stature within the normal range. Actually, there is no consensus whether boys with DP may reach a final height related to the target height [11]. Either the precise and timely diagnosis of CDGP and the elimination of possible pathological growth patterns are useful to an adequate management of CDGP. The classification of pubertal delay is based on a precise anamnestic investigation and a careful examination with the exclusion of either dysmorphic syndromes and systemic diseases. In some cases a physical examination alone may be sufficient.

**Table 1:** Main characteristics of the three groups with absence of pubertal signs at a chronological age of 13 years (females) and of 14 years (males)

	CDGP	FHH	PHH
Bone age f (ys)	< 12	< 12	> 12
Bone age m (ys)	< 13	< 13	> 13
Familar history	yes	no	no
Intercurrent disease	no	yes	no
cryptorchidism, or testes volume ≤ 1 ml	no	no	no/yes
BMI	normal	low	normal/high
Psychological difficulties	no/yes	no/yes	no/yes
FSH	normal/low	normal/low	low
GnRH test	prepubertal	prepubertal	prepubertal
Inibin B	normal/low	normal/low	low
FSH-iB	normal	normal	low
Genetic testing +	no/yes	no	no/yes

*CDGP*: Constitutional delay of growth and puberty; *FHH*: Funtional hypogonadotropic hypogonadism; *PHH*: Permanent hypogonadotropic hypogonadism; *FSH*: Follicle-Stimulating Hormone; *FSH-iB*: FSH stimulated inhibin B concentrations

### Therapeutic approach

The indication of treating CDGP is limited to prepubertal subjects who are older than 14 years of age and have serious psychological distress, mainly correlating to bullying. In girls with CDGP, treatment with a limited dose of estradiol (5-10 mcg daily) for up to 12 months is rare and should led to breast development. Hormonal treatments should be carefully prescribed not to stimulate acceleration in skeletal maturation and not to increase the consequent risk of reduced stature. In fact, in cases of constitutional DP, the best course of action is patience and reassurance. In males a cycle of 50 mg/month of testosterone, increased to 100 mg after 6 months may be offered if psychological problems are exacerbated by the delay [12]. Another possibility is transdermal testosterone administration, beginning with one puff every second day for 3 months, increasing the dosage progressively [13]. During treatment a progressive increase of the testicular volume confirms the diagnosis of CDGP. The treatment should continue until a volume of 12 ml is reached, since at that point the boy can produce a substantial amount of testosterone allowing a normal growth. Therefore, the start of treatment should be individualized depending mainly on the psychological repercussions including low self-esteem, poor school performance, depression and bullying [12]. On the other hand, in hypo- or hyper-gonadotropic hypogonadism long-term hormone substitutive therapy is advised. Girls usually start on a low dose of estrogen administered orally with tablets (5-10 μg/Kg per day) or through transdermal patches every 3-4 days per week, for cycles of 6 months until breast development reaches Tanner III stage. Because of individual variability in the absorption of estradiol, serum estradiol values must be monitored. In 1 year, progesterone is added to facilitate the menstrual cycle and to increase bone density in puberty. Boys usually start with intramuscular injections of 50 mg/month of testosterone for 6 months, rising progressively the dose until an adult dosage (250 mg/month) is reached. However, as for stature, data are not consistent with the indication of growth hormone therapy in increasing adult height in subjects with reduced height and delayed puberty, particularly in the female sex [14]. While, in cases of significant pubertal delay, if no sex steroid hormones treatment is started, acquisition of bone mass can be reduced leading in adulthood to a major risk of future fractures mainly in men [15]. Current studies do not demonstrate that initial testosterone therapy impairs future fertility in boys with PHH; however, if new studies were to demonstrate superiority of early gonadotropin treatment, identifying youth with CHH may guide therapy. Thus, the identification of a generalizable, economic and easily administered diagnostic test for delayed puberty still remains an important endpoint for researchers [9]..

## Conclusion

In conclusions, the ability to make a correct and prompt diagnosis has clinical implications. CDGP subjects have a late but normal puberty, which starts spontaneously. On the contrary, hypogonadic patients do not initiate spontaneous pubertal development. The spontaneous start of puberty by 18 years of age is the gold standard for distinguish CDGP from HH. Paediatricians often cannot differentiate CDGP from isolated HH, until the absence of spontaneous puberty by age 18. The lack of pubertal progression for more than 2 years after spontaneous onset at the appropriate age is indicated in girls by a failure to achieve menarche from the onset of puberty. As for boys it is the failure of testes adult size from 4 mL for more than 5 years. Paediatric Endocrinologists should routinely propose GnRH or GnRH agonist tests as early discriminators to obtain a correct diagnosis quickly and thus avoid anxiety in patients and their families. A detailed personal medical history, including auxological parameters and bone maturation, as well as a precise physical examination are useful in diagnostic approach to an adolescent with DP. Familial history may be used to support the diagnosis of CDGP. Finally, biochemical and haematological parameters may be prescribed to spot some chronic systemic conditions that can present only by delayed growth and puberty.

## Data Availability

At Prof. Mauro Bozzola’s repository.

## Abbreviations

CDGP: Constitutional delay of growth and puberty
DP: Delayed puberty
HH: hypogonadotropic hypogonadism
GH: growth hormone
PHH: Permanent hypogonadotropic hypogonadism
